# *Vibrio cholerae*, classification, pathogenesis, immune response, and trends in vaccine development

**DOI:** 10.3389/fmed.2023.1155751

**Published:** 2023-05-05

**Authors:** David A. Montero, Roberto M. Vidal, Juliana Velasco, Sergio George, Yalda Lucero, Leonardo A. Gómez, Leandro J. Carreño, Richard García-Betancourt, Miguel O’Ryan

**Affiliations:** ^1^Departamento de Microbiología, Facultad de Ciencias Biológicas, Universidad de Concepción, Concepción, Chile; ^2^Programa de Microbiología y Micología, Instituto de Ciencias Biomédicas, Facultad de Medicina, Universidad de Chile, Santiago, Chile; ^3^Instituto Milenio de Inmunología e Inmunoterapia, Facultad de Medicina, Universidad de Chile, Santiago, Chile; ^4^Unidad de Paciente Crítico, Clínica Hospital del Profesor, Santiago, Chile; ^5^Programa de Formación de Especialista en Medicina de Urgencia, Universidad Andrés Bello, Santiago, Chile; ^6^Departamento de Pediatría y Cirugía Infantil, Hospital Dr. Roberto del Rio, Facultad de Medicina, Universidad de Chile, Santiago, Chile; ^7^Programa de Inmunología, Instituto de Ciencias Biomédicas, Facultad de Medicina, Universidad de Chile, Santiago, Chile

**Keywords:** *Vibrio cholerae*, cholera toxin, cholera, diarrhea, oral vaccine, next-generation vaccines

## Abstract

*Vibrio cholerae* is the causative agent of cholera, a highly contagious diarrheal disease affecting millions worldwide each year. Cholera is a major public health problem, primarily in countries with poor sanitary conditions and regions affected by natural disasters, where access to safe drinking water is limited. In this narrative review, we aim to summarize the current understanding of the evolution of virulence and pathogenesis of *V. cholerae* as well as provide an overview of the immune response against this pathogen. We highlight that *V. cholerae* has a remarkable ability to adapt and evolve, which is a global concern because it increases the risk of cholera outbreaks and the spread of the disease to new regions, making its control even more challenging. Furthermore, we show that this pathogen expresses several virulence factors enabling it to efficiently colonize the human intestine and cause cholera. A cumulative body of work also shows that *V. cholerae* infection triggers an inflammatory response that influences the development of immune memory against cholera. Lastly, we reviewed the status of licensed cholera vaccines, those undergoing clinical evaluation, and recent progress in developing next-generation vaccines. This review offers a comprehensive view of *V. cholerae* and identifies knowledge gaps that must be addressed to develop more effective cholera vaccines.

## 1. Introduction

Cholera is an acute, watery diarrheal disease caused by *Vibrio cholerae*, a curved, rod-shaped, motile, Gram-negative bacterium that lives in aquatic environments. Without prompt treatment, cholera can cause severe dehydration and death. Treatment involves administering saline oral rehydration solutions, intravenous fluids, or antibiotics, depending on the severity (1–3). *V. cholerae* is spread from person to person via the fecal-oral route or indirectly through contaminated food and water (3). Cholera is endemic in many regions of Africa and Asia, where seasonal or sporadic outbreaks occur (4–7), predominantly in countries with poor sanitary conditions, such as open defecation, unhygienic food handling, and limited access to safe drinking water (8).

*Vibrio cholerae* is of major public health concern due to its potential to cause pandemics. Since 1817, there have been seven cholera pandemics, with the seventh beginning in 1961 and continuing until today. In 2015, the estimated annual incidence of cholera was 1.3–4 million cases, resulting in 21,000–143,000 deaths (9). However, the notification of cholera cases to the WHO is not mandatory; therefore, it is an underreported disease in many countries (9). For several reasons, the true burden of cholera is underestimated. For instance, it is often difficult to differentiate cholera from other acute diarrheal diseases based on clinical observation. Additionally, diagnostic and epidemiological surveillance laboratories may be deficient or even absent in cholera-endemic areas, thereby limiting accurate etiological diagnosis. It is likely that many cholera-associated cases and deaths do not present to health facilities and are therefore not included in the reports. Added to this, in some countries, there might be disincentives to report cases due to the possible negative impact on tourism and the export industry (10). Recently, the SARS-CoV-2 pandemic has affected cholera surveillance in many regions (11, 12), and there were 65% fewer cases reported to the WHO in 2020 than in 2019 (13). At the same time, preventive measures implemented during the pandemic, such as handwashing, hygiene promotion, social distancing, and banning of large gatherings, likely reduced cholera transmission. The extent to which the SARS-CoV-2 pandemic affected cholera surveillance and epidemiology is currently unknown (14, 15). Thus, cholera remains a leading cause of morbidity and mortality in several developing and resource-poor countries (14).

Cholera is a preventable and treatable disease, and several strategies can be used to control it (Box 1). In 2017, the Global Task Force for Cholera Control proposed an ambitious plan to eliminate endemic cholera in 20 countries and reduce cholera deaths by 90% by 2030 (22). The plan, called “Ending Cholera: A Global Roadmap to 2030,” focuses on strengthening public health systems, improving surveillance for early detection of cholera outbreaks, improving drinking water, sanitation, and hygiene conditions, making oral rehydration treatments more accessible, and increasing vaccination coverage.

**BOX 1 Cholera prevention and control strategies.**

- **Improved sanitation and access to drinking water**: This disease is primarily spread through the consumption of contaminated water or food. Therefore, improving access to drinking water and sanitation facilities can contribute to reducing the risk of cholera transmission (16).
- **Early detection and prompt treatment**: Rapid detection of cholera cases and adequate treatment can reduce the spread of the disease and decrease the number of deaths. Rapid diagnostic tests are useful in this regard (17).
- **Vaccination**: Oral cholera vaccines (OCVs) have been shown to be effective in preventing cholera and should be used as part of a comprehensive cholera control strategy, especially in endemic areas or during outbreaks (18).
- **Health education**: Education campaigns can help to raise awareness about cholera and how to prevent it. These campaigns should include information on proper food storage and preparation, hand washing, and recognizing the signs and symptoms of cholera (19).
- **Strengthening health systems**: A strong health system is crucial for effective prevention, detection, and response to cholera. This requires trained health workers, laboratory capacity, and adequate supplies of vaccines, antibiotics, and oral rehydration solutions (20).
- **Antimicrobial resistance (AMR) surveillance**: Severe cholera is treated with antibiotics, but the emergence of antibiotic-resistant strains can make treatment more difficult. AMR surveillance is essential to ensure appropriate antibiotic use and prevent the spread of resistant strains (21).
- **International cooperation**: Cholera is a global health problem and requires a coordinated global effort. The WHO, along with non-governmental organizations (NGOs) and other international organizations, plays a key role in coordinating efforts to control cholera.

While these strategies can help to control the burden of cholera and prevent large outbreaks, it is important to note that *V. cholerae* will likely never be completely eradicated, as this bacterium is ubiquitous in aquatic environments.

Antibiotic prophylaxis can theoretically prevent both human-to-human and environment-to-human cholera transmissions. Also, some field trials have suggested that chemoprophylaxis has a protective effect among household contacts of people with cholera (23, 24). However, due to the risk of resistance selection, antibiotic prophylaxis for close contacts, as well as for travelers arriving in or departing from cholera-affected areas, is not usually recommended (25).

Efforts and research directed toward the development of cholera vaccines date back more than a century. The first cholera vaccine, a live whole-cell injectable formulation, was developed in 1885 (26). A few years later, killed and attenuated cholera vaccines were reported in 1888 and 1892, respectively (27). Other injectables cholera vaccines were developed throughout the first half of the 20th century. However, all these vaccines had low levels of protective efficacy (PE) and a concerning history of adverse effects (28).

The start of the seventh cholera pandemic in the 1960s and the spread of this disease throughout Asia and Africa led to increased international interest and funding for cholera research, resulting in the development of the first oral cholera vaccine (OCV). It should be noted that current OCVs exhibit variable PE in human populations for several reasons, including the presence of different *V. cholerae* strains in endemic areas, immunization coverage, malnutrition, co-infections, and variations in the gut microbiome (29, 30). Thus, a cholera vaccine that provides broad and long-lasting protection remains elusive.

In this review, we will discuss recent advances in understanding the *V. cholerae* pathogenesis and immunity against cholera, as well as the current status of approved cholera vaccines. Lastly, we discuss how all this knowledge gained could lead to the development of next-generation cholera vaccines.

## 2. *Vibrio cholerae* classification

**Figure 1:**
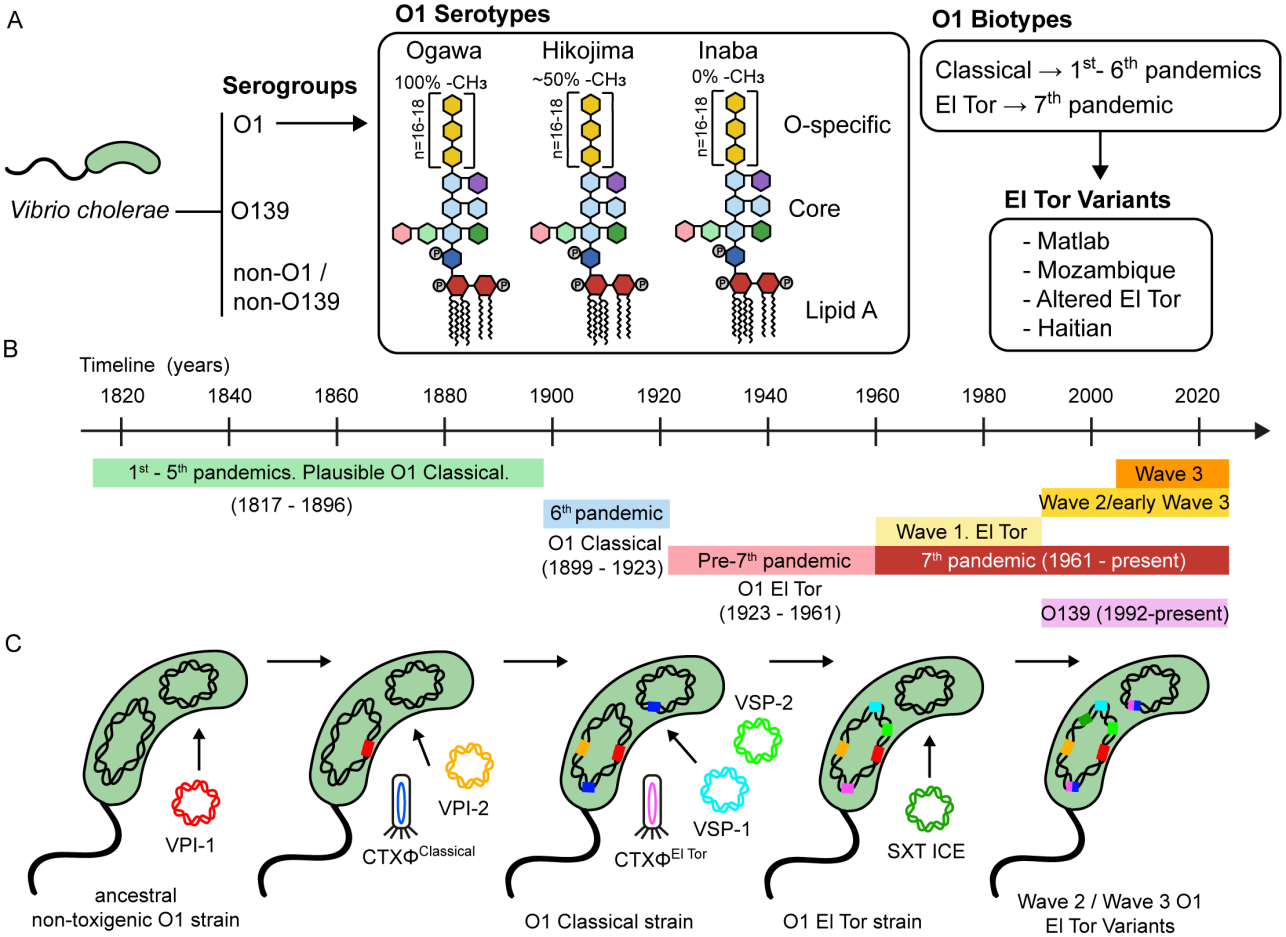
Classification and evolution of *V. cholerae*. **(A)** *V. cholerae* is classified into serogroups based on the composition of the O antigen of LPS. Strains belonging to the O1 serogroup are further divided into three serotypes, namely Ogawa, Hikojima, and Inaba. The LPS of these three serotypes is schematically represented, showing the approximate percentage of methylation of the terminal perosamine. Serogroup O1 is also classified into the Classical and El Tor biotypes, based on phenotypic and genetic markers. Over the past two decades, there has been a growing number of reports on *V. cholerae* strains that possess genetic features from both the Classical and El Tor biotypes, leading to the emergence of hybrid or variant strains. These strains have been linked to several cholera outbreaks worldwide and have contributed significantly to the global burden of this disease. **(B)** Timeline of the history of cholera pandemics. **(C)** A schematic representation of the evolutionary process underlying the development of virulence in serogroup O1. This process is mainly driven by the acquisition of mobile genetic elements, including bacteriophages, genomic islands, integrative and conjugative elements, among others.

*Vibrio cholerae* is divided into more than 200 serogroups determined by the structure of the O-antigen of lipopolysaccharide (LPS) (Figure 1A). Among them, a subset of strains belonging to serogroups O1 and O139 can cause cholera and epidemics due to their ability to produce cholera toxin (CTX). Serogroups that are not O1 and O139, collectively referred to as non-O1/non-O139, typically lack the CTX and cause small gastroenteritis outbreaks, sporadic cases of bacteremia, and wound infections, but they do not cause cholera (31–33). Unlike O1, more than 85% of non-O1 serogroups (including O139) have a capsule that is critical for virulence in extraintestinal infections (34).

Furthermore, O1 strains are divided into three serotypes, designated Ogawa, Inaba, and Hikojima, which are grouped according to the methylation status of the terminal perosamine of the LPS. Ogawa strains are methylated, Inaba strains are unmethylated, and Hikojima strains express both methylated and unmethylated O-antigens. While the Ogawa and Inaba serotypes can co-circulate during epidemics and are capable of interconverting (35), the Hikojima serotype is rare, and evidence indicates that it is an unstable transitional form that results when a strain undergoes serotype switching from Ogawa to Inaba (36).

Biotype is another key classifier of *V. cholerae* O1 strains. Classical and El Tor biotypes can be distinguished according to a set of phenotypic and genetic markers (37, 38). Interestingly, there are some differences in the infection patterns between both biotypes. El Tor strains are more efficient at host-to-host transmission, survive better in the environment and the human gut, and have a higher occurrence of asymptomatic than symptomatic carriers, compared to the Classical strains (39).

## 3. Cholera epidemics and pandemics

It seems that the first five cholera pandemics were caused by Classical biotype strains (1817–1896) (Figure 1B) (40). After this, the sixth cholera pandemic (1899–1923) was caused by the Classical biotype. The Classical biotype was prevalent until the 1960s, but during the pre-seventh-pandemic period (1923–1961), some sporadic outbreaks associated with the El Tor biotype were reported. The ongoing seventh pandemic (1961 to date) is caused by the El Tor biotype (41). Notably, after the emergence of the El Tor biotype, the Classical biotype declined and disappeared by the 1980s, and it is now considered extinct (42).

Eight distinct phylogenetic lineages have been identified based on whole-genome sequencing and genomic analyses of different pandemic strains. The L1 and L3-L6 lineages include Classical strains from the first six pandemics. The L2 lineage includes the El Tor strains of the seventh pandemic (7PET) and is subdivided into three clades (waves 1–3) that represent independent waves of transmission (43). Subsequent analysis reported subclades within individual waves and several transmission events, namely, T1-T12 from African countries, LAT-1 to LAT-3 from Latin America, and T13 from East Africa and Yemen (43–45).

Wave 1 strains were prevalent between 1961 and the early 1990s. During the 1990s, serogroup O139 emerged and caused cholera epidemics in Southeast Asia, but its incidence declined a few years later, and it is now rarely isolated. At the same time, Wave 2 and early Wave 3 strains emerged and replaced Wave 1 strains. Interestingly, many Wave 2 and Wave 3 strains display a mix of phenotypic and genotypic traits of Classical and El Tor biotypes, suggesting that they are genetic hybrids (37). These hybrid strains include the Matlab variants from Bangladesh, the Mozambique variants, the Haitian variants, and the altered El Tor biotype from various parts of the world (46). While Wave 2 strains have waned since the 2000s, Wave 3 strains are now the dominant cause of cholera globally (47).

## 4. Genome and evolution of virulence of *Vibrio cholerae*

The genus *Vibrio* commonly harbors two nonhomologous circular chromosomes, Chr1 and Chr2 (48). The first complete genome sequence of a *V. cholerae* strain was announced for the clinical isolate O1 El Tor Inaba N16961 (49). Genomic analysis of this strain revealed that Chr1 has 2.96 Mb with a 47.7% G + C content, while Chr2 has 1.07 Mb with a 46.9% G + C content. Chr1 contains a large number of genes for essential cellular functions, such as DNA replication, transcription, translation, and cell-wall biosynthesis, as well as virulence genes encoding toxins, adhesins, and surface antigens. By contrast, the Chr2 has fewer such genes and contains a very large integron comprising genes with diverse functions. Comprehensive analysis of both chromosomes revealed the presence of a suite of mobile genetic elements (MGEs), including prophages, genomic islands (GIs), and integrative and conjugative elements (ICEs) (49). Table 1 describes a select list of MGEs that are important in pandemic *V. cholerae* strains.

**Table 1:** Main mobile genetic elements harbored by pandemic *V. cholerae* strains.

Mobile genetic elements	Description
Prophage CTXΦ	It is a filamentous bacteriophage of ∼6.7 kb single-stranded DNA that contains the *ctxA* and *ctxB* genes encoding CTX, as well as the *zot and ace* genes encoding accessory toxins (50).
Prophage TLCΦ	It is a satellite bacteriophage of ∼5.3 kb in size that facilitates stable integration of CTXΦ (51).
*Vibrio* pathogenicity island-1 (VPI-1)	Also known as TCP island, it is ∼41.3 kb in size. It integrates into the Chr1 and contains genes encoding the toxin-coregulated pilus (TCP), the ToxR regulon, and the metalloprotease TagA (52).
*Vibrio* pathogenicity island-2 (VPI-2)	It is ∼57 kb in size. It integrates into the Chr1 and contains several gene clusters, including genes required for the scavenging (Sialidase, *nanH*), transport (*dctPQM*), and catabolism (*nan-nag* region) of sialic acid (53).
*Vibrio* seventh pandemic island-1 (VSP-1)	It is ∼16 kb in size. It integrates into the Chr1 and encodes the dinucleotide cyclase (DncV) enzyme, which is essential for producing intracellular signaling molecule cAMP- GMP. DncV is required for efficient intestinal colonization of the seventh-pandemic strains (54).
*Vibrio* seventh pandemic island-2 (VSP-2)	It is ∼26.9 kb in size. It Integrates into the Chr1 and encodes RNase H1, DNA repair protein, methyl-accepting chemotaxis proteins, and type IV pilus. VSP-2 could be necessary for the evolutionary fitness and epidemic spread of the seventh pandemic strains (55).
SXT integrative and conjugative element (ICE)	It is ∼100 kb in size. It carries multiple antibiotic-resistance genes that confer resistance to sulfamethoxazole, trimethoprim, and streptomycin (56).
Superintegron	Located in the Chr2, it is a large gene capture system of approximately 125 kb, predominantly comprising hypothetical genes, and is proposed as a source of genetic variation (57).

The genomic plasticity of *V. cholerae* and its ability to exchange genes through natural transformation, conjugation, and transduction are hallmarks of this bacterium. Its evolution is continuous due to the acquisition or loss of genomic segments (58, 59). The acquisition of MGEs is known to be the major driver for the evolution of *V. cholerae* virulence and a determinant of genetic divergence between environmental and pandemic strains (60, 61). In this respect, understanding the evolutionary events that lead to the emergence of pandemic clones of *V. cholerae* might provide new approaches for controlling this pathogen.

Chun et al. (62) proposed a hypothetical evolutionary pathway for the emergence of the seventh pandemic *V. cholerae* strains (Figure 1C). According to this model, the diversification of a common ancestral strain occurred through the sequential acquisition of MGEs, likely driven by environmental factors. After acquiring the O1 antigen, an O1 progenitor strain probably acquired the *Vibrio* pathogenicity island-1 (VPI-1) and *Vibrio* pathogenicity island-2 (VPI-2), which are ubiquitous among strains from the sixth (Classical biotype) and seventh (El Tor biotype) pandemics (63). VPI-1 encodes the toxin-coregulated pilus (TCP), which is the receptor for bacteriophage CTXΦ. Thus, transduction by the CTXΦ must have been preceded by the acquisition of VPI-1. The divergence between the Classical and El Tor biotypes was due to the acquisition of distinct bacteriophages CTXΦ and the *Vibrio* seventh pandemic islands (VSP-1 and VSP-2). Several lines of evidence support this. For example, comparative nucleotide sequence analyses have revealed that the CTXΦ from Classical and El Tor biotypes comprise two distinct lineages, indicating that they were acquired in independent events (64–66). In addition, VSP-1 and VSP-2 are consistently found in the O1 El Tor and O139 strains but are predominantly absent in the O1 Classical strains isolated between 1817 and 1923 (40, 59).

Horizontal gene transfer events have also occurred among strains from the seventh pandemic. Unlike Wave 1 strains, Wave 2 and Wave 3 strains contain a self-transmissible integrative conjugative element that carries multiple antibiotic-resistance genes (SXT ICE). The acquisition of SXT ICE likely influenced the population shift from the Wave 1 to Wave 2/3 strains (43). Interestingly, O139 strains that emerged in the 1990s also harbor the SXT ICE (56). In addition, Wave 2 and Wave 3 strains have undergone multiple CTXΦ substitutions and replacements, leading to the emergence of El Tor variant strains (47, 67).

## 5. Pathogenesis of *Vibrio cholerae*

**Figure 2:**
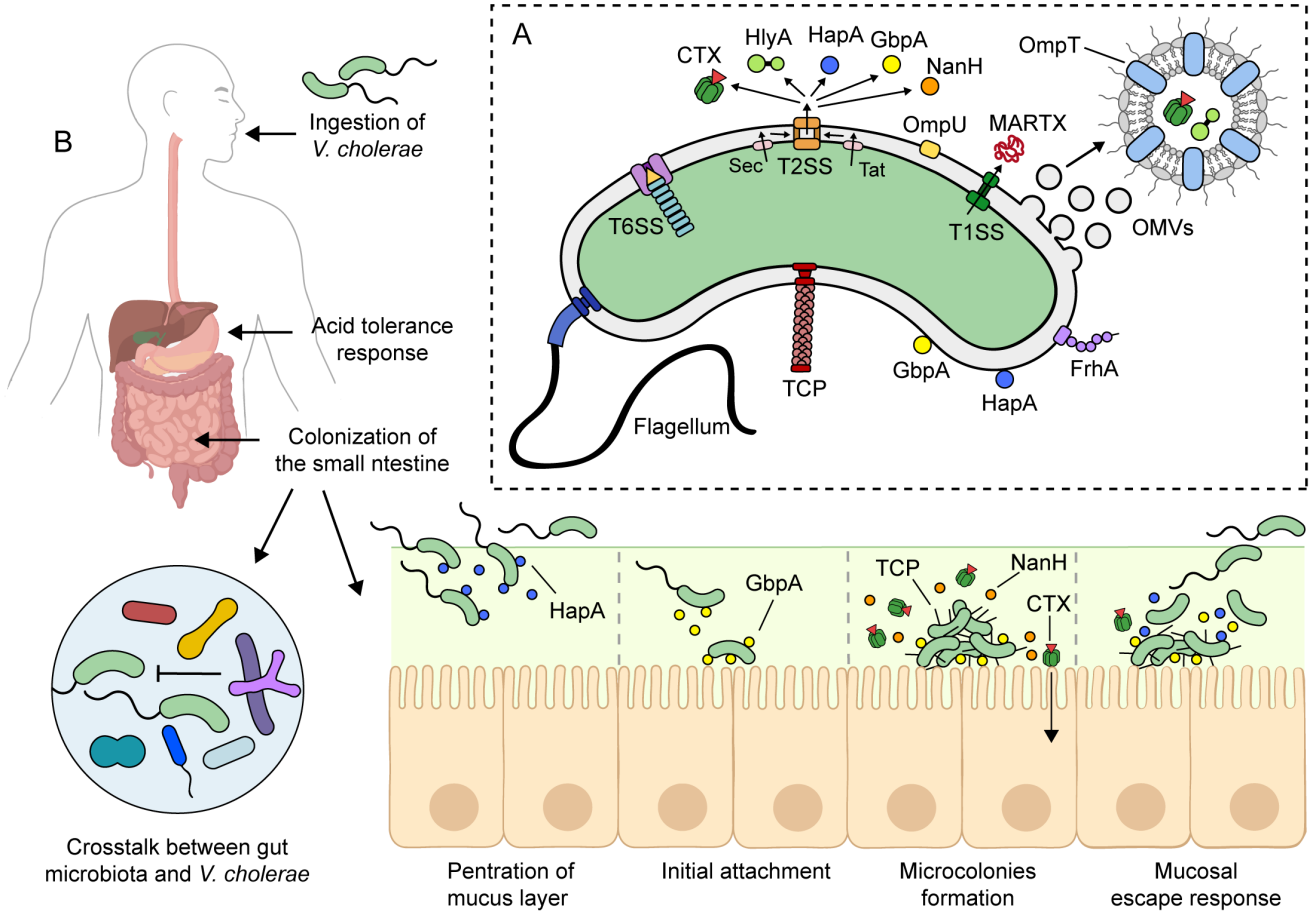
Pathogenesis of toxigenic *V. cholerae*. **(A)** Toxigenic *V. cholerae* produces several virulence factors that contribute to its pathogenesis. The precise pathogenic mechanism is not yet fully understood, but it is widely accepted that it involves the combination of these virulence factors and the ability to colonize and persist in the small intestine. **(B)** Upon ingestion, *V. cholerae* survives the low pH of the stomach *via* an acid tolerance response. In the small intestine, *V. cholerae* uses its flagellum to propel through the mucus layer and reach the epithelial surface. Meanwhile, *V. cholerae* must overcome host immunity and the colonization resistance mechanisms of the gut microbiota. To colonize the small intestine, it expresses virulence factors such as toxin-coregulated pilus (TCP) and cholera toxin (CTX). During infection, other factors such as HapA, GbpA, and NanH are also expressed. For more details on the roles of these virulence factors, please refer to the text. This figure was created using BioRender.com.

In this section, we will review the current understanding of the pathogenesis of toxigenic *V. cholerae* strains, particularly the O1 serogroup. Much of this information has been obtained from *in vitro* assays and challenge experiments in animal models, although some findings have been subsequently confirmed in human infections. Table 2 provides a summary of the main virulence factors of *V. cholerae* that are expressed during infection, and Figure 2A depicts some of these virulence factors.

**Table 2:** Selected virulence factors of *V. cholerae* expressed during human infection.

Virulence factor	Description
Main virulence factors	
Cholera toxin (CTX)	CTX is the main virulence factor in toxigenic *V. cholerae* strains. It belongs to the AB5 family of toxins, which are composed of the catalytic A subunit (CTX-A) and the pentameric receptor-binding B subunit (CTX-B). These subunits are encoded by the *ctxA* and *ctxB* genes located in the filamentous bacteriophage CTXɸ (68). CTX is responsible for the secretory diarrhea characteristic of cholera. It is secreted through the type II secretion system (T2SS) and as cargo within outer membrane vesicles (OMVs) (69–72).
Toxin-coregulated pilus (TCP)	TCP is a type IV pilus with structural similarities to the T2SS. Bacterial aggregation in the form of microcolonies through pilus-pilus interaction with TCP is required to colonize the human intestine. The expression of TCP is coordinately upregulated with that of CTX (73). Furthermore, TCP is the receptor for CTXΦ. Therefore, the evolution of virulence in non-toxigenic *V. cholerae* strains involves the sequential acquisition of VPI followed by CTXΦ (52).
Accessory toxins	
Multifunctional autoprocessing repeats-in-toxin (MARTX) toxin	MARTX toxin is secreted through the type I secretion systems (T1SS). This toxin forms pores in the membranes of target eukaryotic cells and translocates multiple functionally independent effector domains, each of which disrupts a key cellular process. This toxin disrupts the actin cytoskeleton, inhibits phagocytosis, and suppresses innate immune signaling in intestinal epithelial cells (IECs), preventing neutrophil recruitment and bacterial clearance (74, 75).
Hemolysin A (HlyA)	HlyA, also known as Cytolysin (VCC), is a toxin that exhibits vacuolizing and pore-forming activity, resulting in ion leakage and eventual cellular death (76, 77). It is secreted through the T2SS as an inactive 79-kDa pro-hemolysin and undergoes post-translational N- terminal cleavage, mainly mediated by the HapA protease, to form an active 65-kDa toxin (78, 79). HlyA is also secreted in association with OMVs (79). Deletion of the *hlyA* gene reduces virulence in infant mice but has no impact on the rate of mild diarrhea in humans (80, 81).
Zonula occludens toxin (Zot)	It affects the structure of actin microfilaments, leading to increased permeability of epithelial tight junctions (TJ), resulting in the passage of large molecules through a paracellular route (82).
Accessory cholera enterotoxin (Ace)	Ace is an integral membrane protein that alters ion transport, causes accumulation in ligated rabbit ileal loops, and is responsible for mild diarrhea. Ace may cause initial intestinal secretion before CTX acts by stimulating Ca^2+^ − dependent Cl^−^ /HCO3^−^ symporters causing extracellular Ca^2+^ influx (83).
Virulence factors associated with intestinal colonization	
Flagella	*V. cholerae* has a single polar flagellum that is used to penetrate the mucin layer; non-motile (aflagellated) vibrios are significantly less efficient at adhesion and colonization or even avirulent (84–86).
Outer membrane protein U (OmpU)	OmpU confers resistance to bile salts and antimicrobial peptides, playing a key role in the survival of *V. cholerae* in the human intestine (87–89). Moreover, OmpU could play a role in adhesion to the intestinal epithelium (90).
Haemagglutinin/protease (HapA)	HapA is a Zn-dependent metalloprotease secreted through the T2SS as a free protease or in a cell-associated form (73). HapA exhibits several proteolytic activities, including modifying toxins and degrading mucin, fibronectin, and lactoferrin (91). It also acts on TJ-associated proteins, disrupting the paracellular barrier function (92). HapA promotes penetration of the mucosal layer, as well as detachment and spreading of infection along the gastrointestinal tract (93).
ToxR-activated gene- A (TagA)	TagA is a 115 kDa secreted metalloprotease that cleaves mucin glycoproteins and cell-surface glycans, which *V. cholerae* could use as a source of nutrients (94, 95).
Sialidase (NanH)	NanH, also known as neuraminidase, is an extracellular enzyme that catalyzes the cleavage of terminal sialic acid residues from complex carbohydrates on glycoproteins and glycolipids. It is secreted through the T2SS (78). NanH specifically removes sialic acid residues from higher-order gangliosides on the membranes of IECs, exposing GM1 gangliosides, the binding site for CTX (96, 97). Some studies suggest that NanH could promote intestinal colonization as sialic acid residues serve as carbon and energy sources for *V. cholerae* (98).
GlcNAc-binding protein (GbpA)	GbpA is secreted through the T2SS (99). It facilitates attachment to the chitinous exoskeleton of zooplankton as well as mucins covering intestinal epithelial cells. Deleting the *gbpA* gene has been shown to affect intestinal colonization in the infant mouse model (100, 101).
Flagellum-regulated hemagglutinin A (FrhA)	FrhA is a large protein (2,251 amino acids) that contains a type I secretion motif and an RTX-like repeat region at the C-terminus. It mediates binding to erythrocytes, epithelial cells, and chitin and enhances biofilm formation. Deletion of the *frhA* gene affects intestinal colonization in the infant mouse model (102).
Secretion systems	
Type I secretion system (T1SS)	Gram-negative bacteria use the T1SS to secrete proteins in a one-step process using ATP. In *V. cholerae*, T1SS is associated with the secretion of RTX proteins such as MARTX (73).
Type II secretion system (T2SS)	The T2SS shares many structural characteristics with the type IV pilus. Proteins secreted by the T2SS are first translocated to the periplasm by Sec or Tat, where they are assembled to acquire a secretion-competent conformation. *V. cholerae* uses the T2SS to export more than 20 proteins involved in colonization, biofilm formation, and virulence (73, 103). Deletion of TS22 in *V. cholerae* affects growth, biofilm formation, antimicrobial resistance, and cell envelope integrity (73).
Type VI secretion system (T6SS)	The T6SS is a contractile nanomachine resembling a T4 bacteriophage that kills target cells through the contact-dependent translocation of toxic effectors (104). During experimental infection in mice, *V. cholerae* has been found to use T6SS to attack members of the gut microbiota, thereby facilitating colonization (105).

The incubation period of cholera can range from 12 h to 5 days (106, 107). Once ingested, *V. cholerae* must rapidly adapt to the human digestive system (Figure 2B). To accomplish this, the bacterium uses a complex signal transduction network that regulates gene expression in response to different environments and stimuli throughout the gastrointestinal tract.

*Vibrio cholerae* is highly sensitive to low pH, and during passage through the stomach, the vibrios undergo an acid tolerance response (ATR) to both inorganic and organic acid. ATR involves several proteins, including the porin OmpU and the transcriptional regulators CadC and HepA, among others (108–110). Despite the ATR, the number of vibrios reaching the small intestine is reduced. In fact, a high infectious dose (10^8^ bacteria) is required to cause severe cholera in healthy volunteers, while a lower dose (10^5^ bacteria) is sufficient when given with antacids to neutralize stomach acid (28, 111).

Upon reaching the small intestine, the main site of colonization, adaptation to antimicrobial agents, such as bile salts and antimicrobial peptides, is crucial. To achieve this, the bacterium modulates its outer membrane protein (OMP) profile through the activation of a tightly regulated signaling pathway known as the ToxR Regulon (112). In the presence of bile salts, ToxR upregulates the expression of OmpU and downregulates the expression of OmpT, two of the most abundant OMPs of *V. cholerae* (113). The change of the OMP composition also involves removal of OmpT by outer membrane vesicle (OMV) production (114). OmpU and OmpT have distinct channel properties: OmpU is more cation-selective than OmpT, and the bile salt deoxycholic acid blocks OmpT porin activity but not that of OmpU (115, 116). Therefore, OmpU confers resistance to bile salts and antimicrobial peptides, playing a crucial role in the colonization and survival of *V. cholerae* in the small intestine (87, 88). Other intestinal environmental signals, such as bicarbonate, mucin, and osmolarity, also modulate the expression of virulence factors in *V. cholerae* (117–120).

To successfully colonize the small intestine, *V. cholerae* must penetrate a highly viscous mucus layer that is approximately 100–400 μm thick (121), or roughly 30–130 times the size of the bacterium. For this, the vibrios use their flagellum to propel through the mucus layer and reach the epithelial surface (122). It is worth noting that nonmotile vibrios are significantly less efficient at colonization or even avirulent (84). Additionally, the penetration of the mucus layer is facilitated by the hydrolysis of mucins by a group of enzymes, such as HapA, TagA, among others (91, 93–95, 123). Vibrios that fail to penetrate the mucus layer do not colonize the intestinal mucosa and are shed in the feces due to the continuous production and replenishment of mucus (124).

Meanwhile, *V. cholerae* needs to overcome host immunity (see next section) and the colonization resistance mechanisms of the gut microbiota (125). In this respect, mucin activates the *V. cholerae* type VI secretion system (T6SS), which operates as a molecular syringe that kills bacterial competitors through the contact-dependent translocation of toxic effectors (104, 126). In mice, *V. cholerae* T6SS has been shown to attack members of the host commensal microbiota, facilitating intestinal colonization (105). Moreover, T6SS has been suggested as a key mechanism conferring enhanced fitness to pandemic *V. cholerae* strains (127). However, secondary bile acids generated by gut microbiota can inhibit the assembly of the T6SS apparatus (126). Recently, differences in the gut microbiota among individuals have been suggested as a possible explanation for the susceptibility or resistance to cholera (125, 128).

The initial attachment of *V. cholerae* to intestinal epithelial cells (IECs) is likely mediated by the GbpA protein. GbpA is regulated by quorum sensing and is expressed at low cell density (129). Additionally, GbpA stimulates mucin secretion by IECs, which in turn enhances GbpA expression (130). GbpA has been shown to bind mucin, and deletion of its encoding gene decreases intestinal colonization in the infant mouse model (100, 101, 130). Other adhesive factors that could play a role in attachment to the intestinal epithelium are the OmpU and FrhA proteins (90, 102, 131).

After attachment to the intestinal epithelium, *V. cholerae* decreases its motility, proliferates, and forms microcolonies, mostly originating from single vibrio cells (95). Colonizing vibrios express CTX and toxin-coregulated pilus (TCP), which are their main virulence factors. CTX is responsible for the secretory diarrhea characteristic of cholera, while TCP mediates adherence and microcolony formation. Both acidic bile and bicarbonate have been shown to induce CTX and TCP expression via the ToxR regulon (112, 119, 132). Importantly, TCP-deficient mutant strains are unable to colonize animal models and the human intestine (133–135).

**Figure 3:**
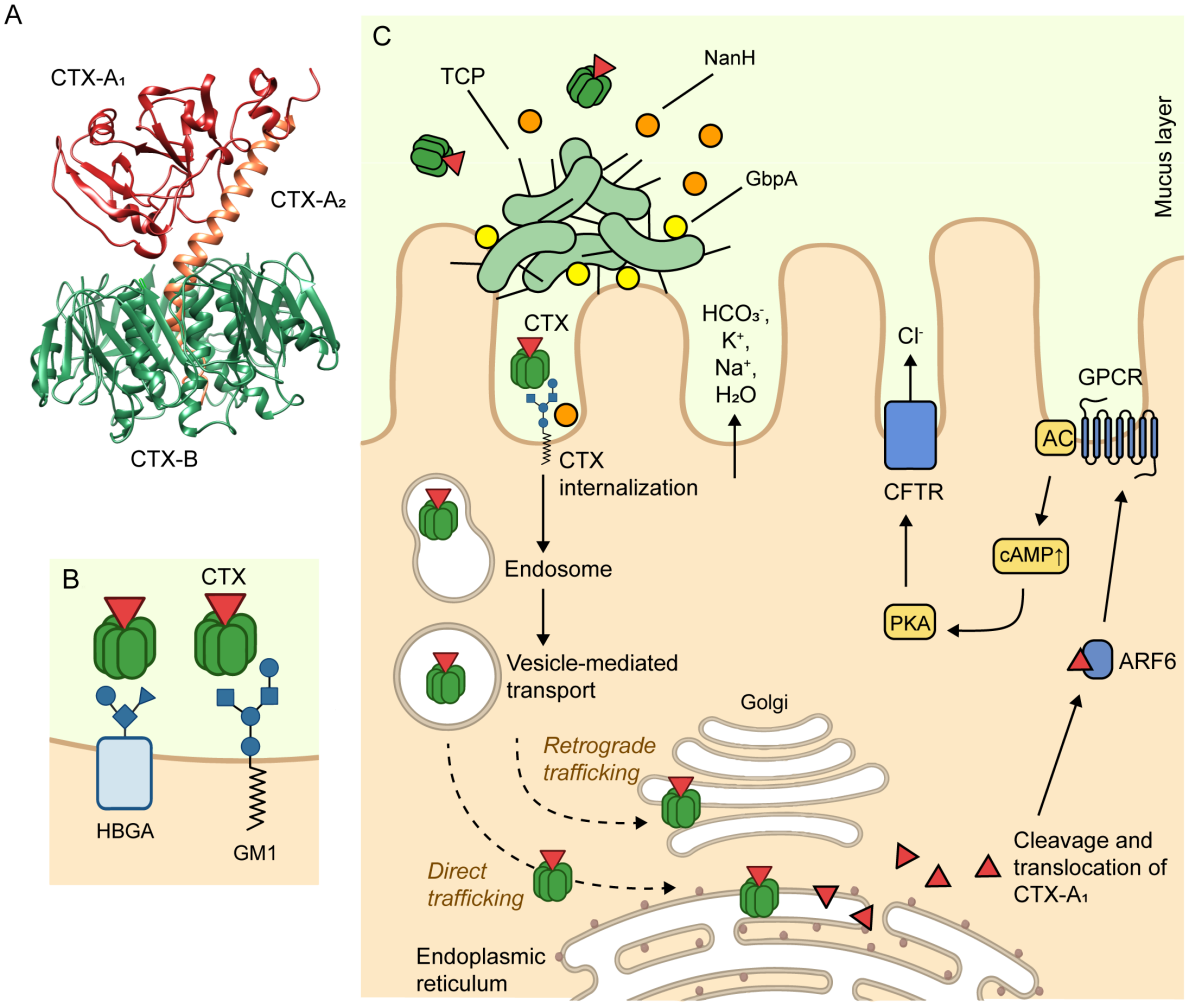
Mechanism of action of cholera toxin. **(A)** The crystal structure of CTX (PDB accession number 1XTC) was determined by Zhang et al. (136). CTX is composed of a heterodimeric CTX-A subunit, which consists of two polypeptide chains, CTX-A_1_ (22 kDa) and CTX-A_2_ (5 kDa), linked by a single disulfide bond. The CTX-A_2_ helical peptide links the CTX-A1 chain to the pentameric CTX-B subunit, which is composed of five identical polypeptide chains (11.6 kDa). **(B)** The CTX-B pentamer specifically binds to GM1 gangliosides (primary receptor) or histo-blood group antigens (HBGAs; secondary binding site) present on the apical side of intestinal epithelial cells, promoting the endocytosis of the toxin. **(C)** The internalization of CTX may occur through clathrin-dependent as well as caveolae- and clathrin-independent endocytosis. Regardless of the mechanism of endocytosis, the CTX is internalized to the early endosomal compartment, trafficked to the Golgi, and then onto the endoplasmic reticulum (ER), where it dissociates into a CTX-A_1_ and a CTX-A_2_/CTX-B complex. Next, the CTX-A_1_ is exported out of the ER to the cytosol, where it is activated by ADP ribosylation factor 6 (ARF6). The ARF6-bound, activated CT-A_1_ subunit, in turn, activates adenylyl cyclase (AC) by catalyzing ADP ribosylation of a G protein-coupled receptor (GPCR). The AC then catalyzes the conversion of ATP to cyclic adenosine monophosphate (cAMP), increasing the intracellular cAMP concentration. This leads to the activation of protein kinase A (PKA), which phosphorylates the cystic fibrosis transmembrane conductance regulator (CFTR) chloride channel proteins, ultimately resulting in the release of electrolytes (Cl^−^, HCO_3_^−^, Na^+^, K^+^) and water into the intestinal lumen, causing the secretory diarrhea characteristic of cholera (137, 138). The figure was created with BioRender.com.

CTX is secreted into the extracellular milieu through the type II secretion system (T2SS) (69). Then, the cellular uptake of CTX occurs via endocytosis, mediated by the binding of CTX-B pentamer to GM1 ganglioside receptors located on the surface of IECs (Figure 3). Of note, NanH cleaves sialic acid from high order gangliosides to release sialic acid and expose the GM1 ganglioside (96, 97). Therefore, NanH promotes the internalization of CTX and its toxigenic effects (139). Although GM1 is considered the primary receptor of CTX, recent studies suggest that CTX-B also binds histo-blood group antigens (HBGAs) at a secondary binding site (140). Additionally, CTX can be released as cargo inside OMVs, which protects the toxin from degradation by intestinal proteases, potentially preserving its toxic effects for longer periods of time (70–72). In particular, CTX-containing OMVs have been shown to be internalized by caveolin-mediated endocytosis in a GM1-independent mechanism that appears to require the presence of OmpU on the vesicle surface (71). After CTX is internalized, cAMP signaling in the IECs is impaired, resulting in a massive release of electrolytes and water into the intestinal lumen, leading to diarrhea (137). The mechanism of action of CTX is described in detail in Figure 3C. Furthermore, other accessory toxins produced by this pathogen can contribute to impaired epithelial barrier function and the development of diarrhea (141). Although 90–95% of infected individuals remain asymptomatic or experience mild symptoms, the remaining 10% develop severe cholera, characterized by profuse watery diarrhea (25). This diarrhea is often described as “rice-water stool” due to its pale, milky appearance (28).

In the late phase of infection, microcolonies of vibrios reach a high cell density, and the nutrients in the intestine decrease. Consequently, vibrios switch from rapid replication to bacteriostasis and downregulate the expression of major virulence factors. Some of them become motile and detach from the epithelial surface moving into the luminal fluid. This process, known as the “mucosal escape response,” is dependent on the general stress response regulator RpoS and the quorum sensing regulator HapR (142–145). Moreover, detachment of vibrios from the intestinal cells is facilitated by the HapA protease, which degrades the GbpA adhesin (129).

Lastly, individuals without effective antibiotic treatment may shed vibrios in their feces for up to 10 days after infection, releasing the bacteria into the environment and increasing the risk of further infections (25). Interestingly, vibrios shed in rice water stool are in a hyperinfectious state (146). These hyperinfectious vibrios are flagellated and highly motile, but most known virulence genes, including those for CTX and TCP, as well as those associated with chemotaxis, are downregulated (147). The exact mechanism for the regulation of the hyperinfectious state remains unknown. In any case, hyperinfectivity is a transient state and is maintained only for a few hours after shedding from the patients (148). Thus, the hyperinfectious state could play a role in the spread of cholera when transmission to another person occurs in a relatively short period of time (149). It is also worth noting that asymptomatic individuals (healthy carriers) are mostly short-term carriers and short-term shedders of vibrios but play an important role in the persistence and transmission of the disease (150).

## 6. Immune response to cholera

Numerous experimental and epidemiological studies have documented that *V. cholerae* infection induces protection against reinfection for at least 3 years in most patients who recover. In this respect, cholera confers greater protection than a subclinical infection (151). However, several factors can affect the immune response against *V. cholerae* and the consequent establishment of immunological memory, including age, nutritional status, blood group, endemicity, co-infections, microbiota, and others (152).

**Figure 4:**
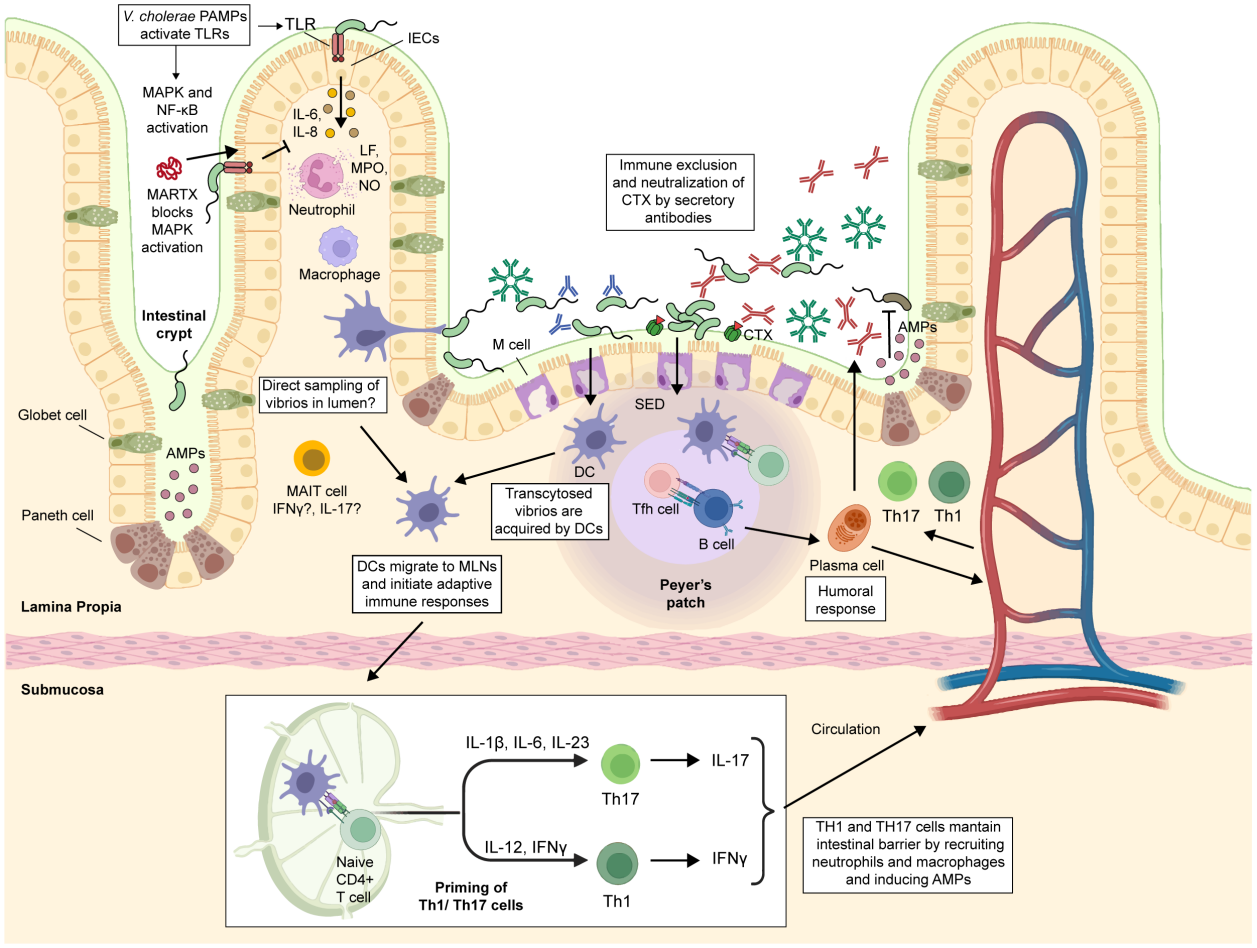
Immune response against cholerae infection. Intestinal epithelial cells (IECs) serve as a physical barrier that limits bacteria to the intestinal lumen. They detect PAMPs such as LPS, flagellin, CTX and OmpU, triggering the secretion of proinflammatory cytokines that recruit innate immune cells such as macrophages, dendritic cells (DCs), and neutrophils. Activated neutrophils increase the inflammation of the intestinal lumen through metabolites such as lactoferrin (LF), myeloperoxidase (MPO), and nitric oxide (NO). M cells take up and transport vibrios from the intestinal lumen to the subepithelial dome (SED) region in Peyer’s patches, where DCs engulf them. Activated DCs migrate to mesenteric lymph nodes, where they produce Th17 or Th1-driving cytokines. Macrophages can also contribute to Th17 or Th1 differentiation through the secretion of IL-23 and IL-6 or IFNγ, respectively. Th1, Th17, and Tfh cells induce B-cell differentiation and expansion. Mucosal-associated invariant T (MAIT) cells are present and highly activated in the lamina propria of the duodenum of cholera patients, but their exact role in the protection against cholera remains to be determined. Secretory antibodies (sIgA and sIgM) prevent vibrios from attaching to the epithelium, blocking their access to the epithelial surface and facilitating their removal through peristaltic activities. Some IgG antibodies could enter the intestinal lumen *via* passive leakage through a damaged and leaky epithelium. The figure was created with BioRender.com.

Although the exact mechanism behind protective immunity against cholera remains largely unknown, our current understanding of *V. cholerae* pathogenesis offers some insight into how this bacterium interacts with the intestinal mucosa and triggers multiple arms of the immune system (Figure 4).

### 6.1. Innate immune response

Cholera has traditionally been considered a noninflammatory diarrheal disease; however, this concept has been re-evaluated, and now it is known that some inflammation occurs during the acute phase of the infection, which is followed by a non-inflammatory convalescent phase (153, 154). In fact, patients with cholera in the acute phase exhibit ultrastructural changes in the duodenal mucosa, such as the widening of the intracellular spaces and alterations of the apical junctional complexes. Moreover, these changes correlate with clinical severity and are characterized by the infiltration of innate immune cells, strongly suggesting an inflammatory response at the site of infection (155).

In the small intestine, IECs play a fundamental role in the defense against enteric pathogens. First, IECs constitute a physical barrier that restricts bacteria to the intestinal lumen. Second, they act as sensors to detect pathogen-associated molecular patterns (PAMPs) and release cytokines that recruit immune cells to the site of infection (156). Mechanistically, during the onset of *V. cholerae* infection, several immunogenic components of this pathogen, such as LPS, flagellins, CTX, and OmpU, can act as PAMPs and be recognized by extracellular and intracellular pattern recognition receptors (PRRs). This triggers the activation of central innate immune pathways, including the myeloid differentiation primary response gene 88 (MyD88), mitogen-activated protein kinase (MAPK), and nuclear factor kappa light-chain enhancer of activated B cells (NF-κB), which in turn activate the secretion of several proinflammatory cytokines (154, 157–162).

For example, *V. cholerae* flagellins induce the expression of IL-1β and IL-8 in IECs by interacting with Toll-like receptor 5 (TLR5) and activating NF-kB and MAPK pathways (159, 161). Likewise, OmpU induces the expression of IL-6, IL-8, and MCP-1 (CCL2) in IECs (163, 164). Moreover, CTX increases intracellular cAMP, leading to IL-6 secretion in IECs (165, 166). These studies were further supported by a transcriptomic analysis of IECs from patients with cholera in the acute phase, where an upregulation of several genes associated with innate immunity was observed (160). Remarkably, activation of the MAPK and NF-κB pathways persisted even 30 days after infection. Furthermore, multiple TLRs, including cell surface TLRs 1, 2, 4, 5, and 6, as well as the endosomal TLRs, including TLR3, TLR7, and TLR8 were upregulated (160).

Among the cytokines mentioned, IL-8 is a potent chemoattractant that recruits polymorphonuclear leukocytes and T cells to the infection site, and stimulates neutrophil degranulation and phagocytosis (167, 168). In addition, the CCL2 chemokine induces the migration of monocytes, dendritic cells, and memory T-cells (169, 170), while IL-6 secretion by IECs activates neutrophil degranulation in the intestinal lumen (166). As a result, innate immune cells, particularly neutrophils, are recruited to the site of infection during the acute phase of cholera (153, 155, 171). Furthermore, a recent study showed that mucosal-associated invariant T (MAIT) cells, an innate-like lymphocyte known to provide immediate effector functions in response to infections, are present and highly activated in the lamina propria of the duodenum of cholera patients (172). Recent evidence suggests that MAIT cells can also provide B cell help and support antibody production at the mucosa level (173); however, further investigation is needed to determine the exact role of MAIT cells in the protection against cholera.

Once neutrophils arrive at the infection site, they express metabolites such as lactoferrin (LF), myeloperoxidase (MPO), and nitric oxide (NO) (153, 174, 175). This may explain the detection of elevated levels of LF and MPO in stools and NO metabolites (NO_2_^−^/NO_3_^−^) in serum of patients with cholera during the acute phase (174, 175). Of note, *V. cholerae* is highly susceptible to the bactericidal action of LF (176, 177). By contrast, in the convalescence phase (~ up to 30 days post infection), an increase of mast cells and eosinophils and their respective effector molecules chymase and IL-3 have been reported (153). Consequently, the activation of proinflammatory signaling pathways, the recruitment of innate immune cells, and their effector functions are fundamental in the initial defense against *V. cholerae*.

Despite the above, *V. cholerae* has some strategies to evade the innate immune response of the host (178). A recent study demonstrated that the accessory MARTX (multifunctional-autoprocessing repeats-in-toxin) toxins secreted by some *V. cholerae* strains can block the MAPK signaling pathway in T84 cells grown *in vitro*. Suppression of MAPK signaling in IECs prevented the recruitment of innate immune cells, and thus this mechanism could protect colonizing vibrios from neutrophil-mediated clearance (74). Importantly, the immunomodulatory effect of these toxins may contribute to the differences in inflammation observed between various *V. cholerae* strains (158). In fact, the current predominant circulating altered El Tor strains lack the MARTX toxins due to a stop codon (179), which could explain in part why these hybrid strains cause a more severe diarrhea and increased intestinal inflammation (158). This raises the question: are innate immune responses in cholera beneficial or detrimental to the host? It is likely that adequate tuning of the innate immune system and a subsequent moderate inflammatory response can be protective against severe cholera.

The gut-associated lymphoid tissues (GALT) play a vital role in developing immunity following natural exposure to enteric pathogens (180). GALT is divided into inductive sites, such as the Peyer’s patches (PP) and mesenteric lymph nodes (MLNs), and effector sites, such as the lamina propria and the intraepithelial lymphocyte compartment (181). Consequently, upon exposure to *V. cholerae*, protective immunity against this pathogen is expected to largely depend on cellular processes that occur in GALT. In fact, in the rabbit ileal loop model, M cells take up and transport vibrios from the intestinal lumen to the subepithelial dome (SED) region in the PP (182). Thus, it is likely that resident DCs and macrophages in the SED become activated and then phagocytose these vibrios during infection in humans.

CTX induces an increase in intracellular cAMP in innate immune cells, leading to the production of IL-1β, TNF-α, and IL-6 (183). In particular, it has been shown that CTX has an immunomodulatory effect on DCs by stimulating their maturation, as well as the expression of MHC class II and costimulatory molecules (184, 185). CTX activates DCs to produce Th17-driving cytokines, including IL-6, which promotes the differentiation of Th17 cells (186, 187). Significantly, CTX also induces the migration of DCs from the SED region to B and T cell zones, where antigen presentation occurs (188). Therefore, the DCs activated by CTX can induce strong humoral and cellular immunity.

Some insights into the role of macrophages against cholera infection have been obtained using the THP-1 human monocyte-like cell line. THP-1 cells exposed to live toxigenic *V. cholerae* upregulate IL-23 expression (189). In another study, THP-1 cells stimulated with *V. cholerae* LPS exhibited increased expression of TNF-α, IL-1β, and MIP-3α through interaction with TLR4 and subsequent activation of the MyD88 pathway (190). It is important to note that both IL-23 and IL-1β are essential for the differentiation of Th17 cells (191, 192).

### 6.2. Adaptive immune response

The subsequent adaptive immune response is complex and involves both humoral and cellular mechanisms. In the acute stage of cholera, studies have shown that lamina propria lymphocytes (LPLs) in the duodenum express significant amounts of IL-6, IL-8, IL-12β, and IL-17 cytokines (162, 193). Later, at day 7 post-infection, cholera induces cellular immune responses in blood compatible with Th1 (IFN-γ) and Th17 (IL-17) profiles (193). Additionally, patients who recover from cholera display an increased percentage of gut-homing CD4^+^ T cells and gut-homing B cells that peak in the circulation 7 days after the infection. Th17 lineage and other IL-17-producing cells play a key role in host defense against bacteria at mucosal surfaces (191), making the Th17 response to *V. cholerae* highly significant. By contrast, the level of gut-homing CD8^+^ T cells reaches its peak in circulation on day 21 (194, 195).

Cholera also triggers follicular helper T (Tfh) cells, which are essential for germinal center formation, affinity maturation, and the development of most high-affinity antibodies and memory B cells (196). A recent study demonstrated that cholera infection in the acute phase induces a significant increase in circulating Tfh cells, which impacts the development of antigen-specific B cells and consequent immunoglobulin production (197).

Considering the pathogenesis of *V. cholerae*, a humoral response capable of neutralizing the CTX, blocking bacterial adherence to the mucosa, and opsonizing the bacteria to mediate their clearance is expected. Consistently, patients who recover from cholera develop systemic IgM, IgG, and IgA antibodies, as well as secretory IgA (sIgA) antibodies, which target several antigens, including CTX-A and CTX-B subunits, LPS, O-specific polysaccharide (OSP), LPS, NanH, flagellins (FlaB, FlaC, and FlaD), TcpA, and HlyA (139, 198–204). Nevertheless, while CTX-specific antibodies confer short-term immunity, the antibacterial (vibriocidal) antibodies are associated with protection against colonization and long-term protective immunity (205).

The best-characterized correlate of protection against cholera is the vibriocidal antibody titer (VAT), which measures the minimum concentration of serum required for antibody-dependent complement-mediated bacterial killing (206). However, VAT is not a comprehensive predictor of long-term immunity. For instance, a specific VAT threshold for absolute protection has not been identified; infection still occurs in a few individuals with very high titers (207). Moreover, there is a lack of mechanistic connection between levels of circulating VAT and prevention of *V. cholerae* colonization at the intestinal mucosal surface level. At the same time, anti-body-dependent complement-mediated bacterial killing is relevant for immunity against systemic infections, it appears to be less important at the intestinal mucosa due to low complement levels at this site (206, 208).

The mechanism by which IgG enters the intestinal lumen is unclear, but it may occur via passive leakage through a damaged and leaky epithelium or through FcRn-mediated epithelial transport (209, 210). Notably, recent studies have demonstrated that IgG anti-OSP contributes to protection against cholera by inhibiting the motility of *V. cholerae*, potentially limiting its access to the apical surface of the intestine (211). It is also possible that anti-OSP sIgA may contribute to protection by inhibiting motility and trapping the bacteria at the mucosal surface (1, 212). Even in the absence of circulating anti-OSP antibodies, OSP-specific memory B cells may maintain protective immunity by rapidly expanding and differentiating into plasmablasts upon antigen exposure (213). Furthermore, a recent study showed that patients with cholera develop IgG, IgA, and IgM antibodies against NanH, and that plasma responses targeting this antigen correlate with protection (214). The protective role of other antibacterial antibodies against cholera is currently unknown.

Thus, this body of studies demonstrates that cholera infection stimulates innate cells at the site of infection, primarily neutrophils and their corresponding effector molecules. The subsequent adaptive response is characterized by Th1, Th17, and Tfh CD4^+^ cells, which shape the antibody production targeting the CTX and various surface-exposed antigens. However, there are currently many knowledge gaps in understanding how these immunological processes occur. In this regard, to develop a highly effective cholera vaccine, it may be necessary to mimic these immune responses. As such, progress should be made in understanding the differences between the immune response triggered by *V. cholerae* infection and that induced by vaccination. This could pave the way for the development of the next generation of cholera vaccines.

## 7. Current vaccines licensed worldwide or with restricted license

The knowledge gained on immunity against *V. cholerae* has not only facilitated the development of current cholera vaccines, but also highlighted the possibility of developing novel vaccines that provide broader and longer-lasting protection. In this section, we will briefly review licensed cholera vaccines, while subsequent sections will focus on candidate vaccines currently undergoing clinical and preclinical evaluation.

In the 1960s, several large field studies conducted in Asian countries showed that injectable killed whole-cell cholera vaccines had modest efficacy and a high rate of adverse reactions, such as fever, local pain and swelling (215). Subsequently, interest shifted to exploring the potential of oral vaccination, which led to the development of the OCVs. Oral vaccines mainly interact with the immune system through the Waldeyer’s tonsillar ring in the oral cavity or via the PP in the small intestine. Compared to vaccines administered by parenteral routes, oral vaccines have been found to induce stronger immune responses at the intestinal mucosa level, partly via antigen-specific sIgA (216). However, oral vaccines face some challenges, including the potential degradation of acid-labile antigens in the stomach, the lack of licensed oral adjuvants for human use, and the difficulty of their release at mucosal immune inductive sites (217).

At present, four OCVs based on killed whole cell vibrios are prequalified by the WHO (meaning that they can be purchased by United Nations agencies): Dukoral^®^, Shanchol^™^, Euvichol, and Euvichol-Plus (Table 3 and Box 2) (217).

**Table 3:** Licensed cholera vaccines.

Vaccine	Manufacturer	Status	Composition	Immunization schedule	Duration of protection
Dukoral^®^	SBL vaccin, Sweden.	Licensed in 1991 in Sweden, and in more than 60 countries since then. WHO prequalification in 2001.	Monovalent vaccine containing heat- or formalin- killed strains of *V. cholerae* O1 (Classical Inaba strain Cairo 48, Classical Ogawa strain Cairo 50, and El Tor Inaba strain Phil 6,973), along with 1 mg rCTB.	Oral administration. For individuals aged 2 years and above. Children aged 2–5 years require 3 doses given 7–14 days apart, with a booster recommended after 6 months. Individuals aged 5 years and older require 2 doses given 7–14 days apart, with a booster recommended after 2 years.	Offers protection for 6 months to 2 years.
mORC-Vax^™^	VaBiotech, Vietnam.	Licensed in 1997 in Vietnam for local use only. Not WHO prequalified.	Bivalent vaccine containing heat- or formalin- killed strains of *V. cholerae* O1 (Classical Inaba strain Cairo 48, Classical Ogawa strain Cairo 50, and El Tor Inaba strain Phil 6,973) and the formalin-killed *V. cholerae* O139 strain 4260B.	Oral administration. For individuals aged 2 years and above. Two doses given 14 days apart. There is no booster recommendation from the manufacturer.	Offers protection for at least 3 years.
Shanchol^™^	Sanofi-Shantha Biotechnics, India.	Licensed in 2009 in India, and in 28 countries since then. WHO prequalification: 2011	Same composition as mORC-Vax^™^.	Oral administration. For individuals aged 1 year and above. Two doses given 14 days apart. There is no booster recommendation from the manufacturer.	Offers protection for at least 3–5 years.
Euvichol^®^ / Euvichol-Plus^®^	Eubiologics, Republic of Korea.	WHO prequalification for Euvichol in 2015 and Euvichol-Plus in 2017.	Same composition as in Shanchol^™^.	Oral administration. For individuals aged 1 year and above. Two doses given 14 days apart. There is no booster recommendation from the manufacturer.	Not available.
OraVacs^™^	Shanghai United cell Biotechnology, China.	Licensed in China and the Philippines. Not WHO prequalified.	Dry formulation enteric-coated capsule vaccine containing a composition similar to Dukoral^®^.	Oral administration. For individuals aged 2 years and above. Three capsules taken on days 0, 7, and 28.	Not available.
Cholvax^™^	Incepta, Bangladesh.	Licensed in 2020 in Bangladesh. Not WHO prequalified.	Same composition as in Shanchol^™^	Oral administration. For individuals aged 1 year and above. Two doses given 14 days apart. There is no booster recommendation from the manufacturer.	Not available.
CVD 103-HgR (Vaxchora^™^)	PaxVax Inc., US.	Licensed in 2016 in USA, and in 2020 in Europe. Not WHO prequalified.	Live, attenuated *V. cholerae* O1 Classical Inaba strain CVD 103-HgR, a derivative of 569B.	Oral administration. For individuals between 2 and 64 years of age. Single dose (4 × 10^8^ to 2 × 10^9^ CFU).	Offers protection for 6 months.

**BOX 2 Advantages and limitations of killed OCVs.**Killed OCVs possess several characteristics that make them effective in combating cholera:

- **Safety**: These vaccines have been proven safe, with only minor side effects reported.
- **Easy administration**: They can be easily administered in mass vaccination campaigns and in settings where injection-based vaccines may be logistically difficult to implement.
- **Cost-effectiveness**: Killed OCVs are relatively inexpensive, making them accessible to populations in resource-limited areas where cholera is prevalent.
- **Herd immunity**: OCVs not only protect the individuals who receive them but also create herd immunity, which can help to decrease transmission of the disease in the community (218, 219).

While killed OCVs offer several benefits as a tool for controlling cholera, they also have some limitations:

- **Limited effectiveness**: Their effectiveness can vary depending on the vaccinated population and the level of cholera transmission in the area. The protection provided by OCVs is short-term and decreases over time.
- **Limited immune response**: These vaccines do not contain live bacteria; thus, the immune response elicited may differ from that triggered by a natural cholera infection. This difference may result in a different pattern of immune response and antibody production, which can affect the duration and quality of the immunity provided.
- **Cold chain requirements**: They must be stored at a specific temperature range (typically between 2 and 8°C) to preserve their immunogenic properties, which can be challenging in areas with limited infrastructure.
- **Requirement for multiple doses**: They require at least two doses to provide adequate protection, which can be a barrier to achieving high coverage in some settings.
- **Limited role in outbreaks**: They do not provide immediate protection against cholera and are not intended to replace other control measures.

Dukoral^®^ was licensed in 1991 and since then has been distributed in over 60 countries. It is a monovalent vaccine composed of a mixture of three heat- or formalin-inactivated *V. cholerae* O1 strains (Classical Inaba strain Cairo 48, Classical Ogawa strain Cairo 50, and El Tor Inaba strain Phil 6973) along with the recombinant CTX-B (rCTX-B) subunit. The vaccine is free of the CTX-A subunit due to its toxicity. A sodium bicarbonate buffer is also added to the formulation to prevent the degradation of rCTX-B by gastric acid. This vaccine can be administered to individuals over 2 years of age and requires at least two doses for optimal efficacy. The protective efficacy (PE) of this vaccine has been demonstrated in several field trials in different countries, achieving over 80% protection in the first 6 months and gradually decreasing thereafter, ultimately providing negligible protection after 2 years. No significant severe adverse effects were attributed to this vaccine (220–222). Further analyses of volunteers vaccinated with Dukoral^®^ revealed that this formulation induces high levels of specific sIgA antibodies and IFN-γ production in the duodenal mucosa (223). Notably, Dukoral^®^ also provides significant protection for 3–9 months (PE: 67%) against diarrhea caused by enterotoxigenic *E. coli* (ETEC) producing heat-labile toxin (LT). This cross-protection is due to the cross-reactivity between the CTX-B subunit and LT (224).

In the late 1980s, the technology for manufacturing Dukoral^®^ was transferred from Sweden to Vietnam for local production. This vaccine contained the same Dukoral strain composition, but the rCTX-B subunit was removed to simplify manufacturing, reduce costs, and improve stability. In 1992, the O139 epidemic in India and Bangladesh led to the addition of a killed O139 strain. This formulation was initially licensed in Vietnam as ORC-Vax^™^ and later after its modification as mORC-Vax^™^. It should be noted that the incorporation of the O139 component did not affect the responses to the original Dukoral components; instead, anti-O1 and anti-O139 antibodies were induced in serum and the intestinal mucosa (225, 226). However, the National Regulatory Agency (NRA) of Vietnam at that time did not have WHO approval, which limited the international use and WHO prequalification of this Vietnamese OCV. To make the vaccine available for international use, the manufacturing technology was transferred from Vietnam to Shantha Biotechnics in India, which had a WHO-approved NRA (195, 227). The PE of this vaccine was evaluated in a trial conducted in Kolkata, demonstrating that a two-dose immunization schedule provides an overall 65% protection over a five-year observation period. In 2009, this vaccine was licensed in India as Shanchol^™^, and WHO prequalified it in 2011 (228, 229).

The manufacturing technology of Shanchol^™^ was later transferred to Eubiologics in Seoul, Republic of Korea, resulting in the production of Euvichol^®^, which has an identical composition to Shanchol^™^. Studies in different countries have shown that Euvichol^®^ and Shanchol^™^ elicit similar vibriocidal antibody responses and have comparable safety profiles. Euvichol^®^ received licensure and WHO prequalification in 2015. Euvichol-Plus^®^ is an improved vaccine that utilizes plastic tubes instead of conventional glass vials, providing better conditions for storage, transportation, and administration. This change has facilitated the delivery of this vaccine in emergency situations or humanitarian campaigns. Euvichol-Plus^®^ received WHO prequalification in 2017 (230).

Two killed OCVs are licensed in some countries but are not WHO-prequalified. OraVacs^™^ is a dry formulation enteric-coated capsule vaccine containing a composition similar to Dukoral^®^. It is licensed in China and the Philippines (231). Cholvax^™^ is licensed in Bangladesh for use in the national cholera control program and has demonstrated safety and immunogenicity comparable to Shanchol^™^ (232).

The OCVs have achieved an important milestone in public health by providing herd immunity in vaccinated communities, thereby reducing person-to-person transmission (218, 219). In addition, the accumulation of evidence regarding the safety and efficacy of these vaccines has led the WHO to recommend their mass use as a preventive strategy in cholera-endemic areas, as well as a response measure during cholera outbreaks. Consequently, the WHO established the global OCV stockpile in 2011, which received support from the Global Alliance for Vaccines and Immunizations (Gavi Alliance) in 2014 (233). The main objectives of the OCV stockpile are to store and provide cholera vaccines during outbreaks and humanitarian campaigns, among other measures to control this disease. Presently, the OCV stockpile primarily uses Euvichol-Plus as its main formulation.

Despite their importance and usefulness, killed OCVs have several limitations. First, the PE of these vaccines is low (~42%) in children under the age of five, who are most vulnerable to the long-term effects and higher mortality associated with cholera (228). Second, they require multiple doses to achieve a high level of protection, which increases economic costs and the time required to elicit immunity. In fact, with a single dose, PE is only 8% for those under the age of five and 57.5% for those over the age of five (234, 235). Third, PE is short-term since it begins to decrease after 6 months and practically disappears after 3 or 5 years. In some sense, these limitations may be intrinsically related to the nature of killed vaccines. For instance, the *in vitro* cultures used to grow the vibrios included in these formulations do not reproduce host conditions and some important antigens may not be expressed. This is the case of the TcpA antigen, which is absent in the killed OCVs (236). Moreover, the formalin and heat treatment used to kill the bacteria may destroy or alter epitopes (237). Ultimately, killed vaccines are unable to mimic natural infection, so immune stimulation may be different from what is needed to achieve long-lasting protection.

Live attenuated OCVs have the potential to overcome many of the intrinsic limitations of killed OCVs. For instance, live attenuated vibrios closely mimic natural infection, and thus, they may trigger immune responses in the GALT, with the potential to target antigens which are only expressed *in vivo* during infection (199). Moreover, live attenuated OCVs may require a single dose, reducing the time required to achieve significant PE; this is particularly advantageous for individuals requiring travel at short notice to areas where an outbreak is occurring or where cholera is endemic (238).

Currently, only one live attenuated OCV is available, named Vaxchora^™^, which is approved in the United States and Europe for travelers visiting regions where cholera is endemic (238). The approval of Vaxchora^™^ in other markets is pending. This vaccine is based on the *V. cholerae* strain CVD 103-HgR, serogroup O1, serotype Inaba, classical biotype, which is derived from the strain 569B. The CVD 103-HgR strain is genetically modified and contains a deletion of the *ctxA* gene and an insertion of the Hg++ resistance gene to enable differentiation of the vaccine strain from the wild type (239). Although the CVD 103-HgR strain cannot produce active CTX, it can synthesize the CTX-B subunit and the TcpA antigen, and colonize the small intestine transiently (199). The initial CVD 103-HgR formulation was introduced in 1993, and since then, it has been manufactured by various companies and known by other trade names such as Orochol^®^, Mutacol^®^, and Orochol-E^®^.

The effectiveness of the CVD 103-HgR vaccine was initially evaluated in four experimental challenge studies between 1987 and 1999, where the PE against severe diarrhea was 92.7, 95.4, 79.0, and 67.6% (239). Moreover, this vaccine can elicit a significant VAT 10 days after immunization, but the duration of protection has not been fully determined (240, 241). However, conflicting results were obtained in two field studies conducted in North Jakarta, Indonesia, between 1993 and 1997 (242), and on the island of Pohnpei, Micronesia, during an outbreak in 2001 (243). The PE obtained in the Indonesian study was only 14%, whereas in the Micronesian study, it was 79.2%. The poor performance of this vaccine in the Indonesian study was attributed to a lower-than-expected cholera incidence (242). Thus, the effectiveness of Vaxchora^™^ in cholera-endemic areas remains unclear.

Additionally, several factors have limited the use of the CVD 103-HgR vaccine beyond the traveler’s market, including possible toxigenic reversal, high cost, and the requirement of a cold chain (−25 to −15°C) (244). For further details beyond what is provided here on the CVD 103-HgR vaccine and the history of its development, the reader is referred to recent comprehensive reviews (238, 239, 244).

## 8. Vaccines candidates in clinical development

Much work has been done in recent years to improve the manufacturing process of killed OVCs, to enhance their stability, and to further reduce costs. An example of this is Hillchol^®^ (245), which was developed by Bharat Biotech International in India. Hillchol^®^ is based on the formalin-killed *V. cholerae* O1 Hikojima strain MS1568 (Table 4). The MS1568 strain is a derivative of Phil 6973 strain, which is a component of Shanchol^™^. It has a partially inactivating mutation in the *wbeT* gene that is responsible for LPS methylation, which differentiates the Ogawa and Inaba serotypes; thus, this strain expresses ~50% of both LPS. As a result, Hillchol^®^ requires a single-strain manufacturing process that is less expensive than other killed OCVs but still maintains a mixed O1 antigen composition (36). Hillchol^®^ completed a phase I/II study evaluating its safety, tolerability and immunogenicity. The study demonstrated that it is not inferior to Shanchol^™^ in individuals of different age groups residing in a cholera-endemic region (246). In August 2022, Hillchol^®^ began a phase III study (Clinical Trial NCT 05507229).

**Table 4:** Cholera vaccine candidates under clinical evaluation.

Vaccine	Manufacturer	Status	Composition	Immunization schedule	Duration of protection
Hillchol^®^	Bharat Biotech International Ltd., India.	Completed phase I/phase II clinical safety and immunogenicity study. Phase III clinical study underway (Clinical Trial NCT 05507229).	Monovalent vaccine containing formalin-killed whole cell of recombinant *V. cholerae* O1 El Tor Hikojima strain MS1568, which expresses ~50% each of Ogawa and Inaba LPS.	Oral administration. For individuals between 1 and 45 years of age. Two doses (under study).	Not available.
CholeraGarde^®^ (Peru-15)	Vaccine Technologies Inc., USA.	Completed phase I/phase II clinical safety and immunogenicity studies, last reported in 2015.	Live, attenuated, non-motile, *V. cholerae* O1 El Tor Inaba strain C6709 (∆CTXΦ *ctxB*::rec*A*).	Oral administration. Single dose (Up to 1 × 10^9^ CFU) for healthy adults, children above aged 9 months, and in HIV-positive adults (aged 18–45 years).	Not available.
Vax-COLER^®^ (Cuban 638)	Finlay Institute, Havana, Cuba.	Completed phase I/phase II clinical safety and immunogenicity study, last reported in 2011.	Live, attenuated *V. cholerae* O1 El Tor Ogawa 638 (∆CTXΦ *hapA*::*celA*).	Oral administration. Single dose (2 × 10^9^ CFU) for individuals between 18 and 50 years of age.	Not available.
VA 1.3 / VA 1.4	Shantha Biotech, India.	Completed phase I/phase II clinical safety and immunogenicity study, last reported in 2014.	Live, attenuated, non-toxigenic *V. cholerae* O1 El Tor Inaba (∆*hlyA*::*ctxB*).	Oral administration. Single and double dose (1.9 × 10^9^ CFU) for individuals between 18 and 60 years of age.	Not available.
Panchol (HaitiV)	Harvard University, USA.	Phase 1 clinical study underway (Clinical Trial NCT 05657782).	Live, attenuated *V. cholerae* O1 El Tor Ogawa strain HaitiV, with nine genetically engineered mutations.	Oral administration. Single dose. CFU concentrations under study: log10 values 6, 7, 8, 9 and 10.	Not available.
OSP:rTTHc	Eubiologics Ltd., South Korea, and Harvard University, USA.	Phase 1 clinical study underway (Clinical Trial NCT 05559983).	Conjugated vaccine candidate containing Inaba or Ogawa OSP linked to recombinant tetanus toxoid heavy chain fragment (rTThc), with or without aluminum phosphate adjuvant.	Immunization schedule under study: two doses of 5, 10, and 25 μg, with or without aluminum phosphate adjuvant, administered intramuscularly 4 weeks apart.	Not available.
MucoRice-CTB (IMSUT-MR1501)	University of Tokyo, Japan.	Completed phase 1 clinical study (UMIN Clinical Trials Registry UMIN000018001)	Oral rice-based vaccine expressing CTX-B subunit.	Oral administration. 6 g once every 2 weeks for 8 weeks (for a total of 4 doses).	Not available.

Over the past three decades, several live attenuated OCV candidates have been developed. However, only four of them have progressed to clinical trials. The oldest among them, CholeraGarde^®^ (Peru-15), was reported in 1995. It is based on a *V. cholerae* O1 El Tor Inaba strain derived from the C6709 strain, which was isolated in Peru in 1991. The Peru-15 strain is attenuated due to a deletion of the CTXΦ prophage and a spontaneous mutation that affects motility. It also has an insertion of the *ctxB* gene in the *recA* gene for the constitutive expression of the CTX-B subunit. Since the *recA* gene is required for homologous recombination, the Peru-15 strain has a reduced capacity for horizontal gene transfer (247, 248). CholeraGarde^®^ was shown to be safe and immunogenic in phase I/II studies conducted in the United States, Bangladesh and Thailand (249–252). In challenge studies, a single dose of this formulation demonstrated a PE of 100% against moderate and severe diarrhea. Additionally, only a small percentage of individuals (7%) developed mild diarrhea after challenge (253). The last clinical trial of this vaccine candidate was reported in 2015, and it is unclear whether it will be evaluated in a phase III study.

Another live attenuated OCV candidate, Vax-COLER^®^ (Cuban 638), was reported in 1999. It is based on the *V. cholerae* El Tor Ogawa strain 638, which is derived from the C7258 strain isolated in Peru in 1991. The 638 strain is attenuated due to the deletion of the CTXΦ prophage and an insertion of the *Clostridium thermocellum* endoglucanase A (*celA*) gene into the hemagglutinin/protease (*hapA*) gene (254). Vax-COLER^®^ has been shown to be safe and immunogenic in phase I/II studies conducted in Cuba and in a cholera endemic area in Maputo, Mozambique (254–256). It has also been found to provide protection against a challenge with the *V. cholerae* O1 El Tor strain 3,008 (257). However, there is currently no available information on whether this vaccine candidate will be evaluated in a phase III study.

A third live attenuated OCV candidate is VA1.4, which was initially reported in 1999 as VA1.3 (258). The VA1.3 is a non-toxigenic *V. cholerae* O1 El Tor Inaba strain with an insertion of the *ctxB* gene (under the control of the *ctx* promoter) into the *hlyA* gene. This strain naturally lacked the CTXΦ prophage and has proven to be non-reactogenic in a rabbit ileal loop assay. In 2009, a phase I/II study conducted in a cholera-endemic area in Kolkata, India, showed that the VA1.3 strain is safe and immunogenic (259). A later version of this vaccine candidate is the VA1.4 strain, which is identical to VA1.3, except for the absence of an ampicillin resistance gene. In 2014, a phase I/II study conducted in Kolkata, India, showed that VA1.4 is also safe and immunogenic (260). This vaccine candidate was evaluated with a two-dose schedule of 1.9 × 10^9^ CFU, but no additional benefit was observed after the second dose. Currently, there is no information available regarding a phase III study for this formulation.

The fourth and most recent live attenuated OCV candidate is PanChol (HaitiV), which was developed in 2018 in the USA (261). HaitiV is derived from a variant O1 El Tor Ogawa strain isolated during the 2010 Haiti outbreak. The HaitiV strain has several genetic modifications that make it avirulent and resistant to reversion, but it maintains the ability to colonize the intestine and induce immune responses. These genetic modifications include deletions of: (i) the entire CTXΦ and its boundaries encoding the MARTX toxin (*rtxABCDE*) genes; (ii) the *hupB* gene required for episomal maintenance of CTXΦ; (iii) five flagellin subunits (*flaA-E*) genes; (iv) a region of DNA containing resistance genes for the antibiotics trimethoprim (*dfrA*), sulfamethoxazole (*sul2*), streptomycin (*strAB*), and chloramphenicol (*floR*); and (v) the *recA* gene involved in gene acquisition by homologous recombination. In addition, HaitiV has an insertion of the *ctxB* gene (under the control of the *htpG* promoter) in the neutral locus N900_11550. To prevent toxigenic reversion, the HaitiV strain also encodes a CRISPR/Cas9 system targeting the *ctxA* gene.

It should be noted that oral administration of HaitiV in animal models has demonstrated a protective effect within 24 h post-vaccination against a lethal dose of the parent *V. cholerae* strain HaitiWT (261, 262). This rapid protection was achieved before the induction of any adaptive immune response, suggesting that HaitiV exhibits a probiotic-like activity. However, it is unclear whether this “probiotic” effect is specific to HaitiV or also present in other live attenuated OVCs. Moreover, immunization of mice with this vaccine candidate was well-tolerated and immunogenic, triggering humoral responses consisting of anti-OSP and anti-CTX-B IgM, IgG, and IgA antibodies. In December 2022, PanChol began a phase I study for safety, tolerability, and immunogenicity in healthy volunteers (Clinical Trial NCT05657782).

As previously mentioned, protection against cholera is mainly serogroup-specific. Furthermore, the generation of anti-OSP antibodies is a common immune response elicited by various cholera vaccines, and these antibodies have been associated with protection in both animal models and in humans. This has been the rationale for the use of LPS and the O-antigen as a target for the development of cholera vaccines. In this regard, vaccine candidates based on the O-antigen conjugated with protein carriers are an interesting alternative to OCVs.

One of the first cholera conjugate vaccine candidates was prepared by binding the detoxified (deacylated) LPS (DeA-LPS) with the CTX (263). Subsequent evaluation of the DeA-LPS-CTX conjugate in a phase I study in adult volunteers showed that it was immunogenic by eliciting vibriocidal (anti-LPS) antibodies and IgG anti-CTX antibodies (264). However, this vaccine candidate was not further evaluated.

More recently, cholera conjugate vaccine candidates were developed by binding the Inaba or Ogawa OSP with the recombinant tetanus toxoid heavy chain fragment (rTThc). Preclinical evaluation of the OSP: rTTHc conjugates has shown that they are immunogenic and protective in mice (265, 266). Interestingly, a combined vaccination approach which includes an oral priming with Vaxchora^™^ followed by a parenteral boost with the OSP: rTTHc conjugate resulted in increased immune responses in mice (267). In 2021, the OSP: rTTHc conjugate candidate was produced in a scalable manner, and the addition of aluminum phosphate adjuvant increased the OSP-specific immune responses in mice (268). In September 2022, the OSP: rTTHc conjugate vaccine began a phase I study primarily to determine the safety of the dose range with or without aluminum phosphate adjuvant, and secondarily to assess humoral immune responses in the nonendemic population, which will guide the selection of future doses (Clinical Trial NCT 05559983).

Plant-based vaccines represent a step toward new vaccinology technologies and oral vaccination. These innovative vaccines have some advantages over classical vaccines, including the long-term preservation of antigenic proteins without the need for a cold chain, resistance to digestion in the stomach, lower cost, increased safety, and scalability (269). Potato, tomato, and rice are attractive antigen-expressing plants that have been used as a platform for the development of candidate vaccines against some infectious diseases in animals and humans (269–271). Notably, the expression of CTX-B subunit oligomers has been reported in transgenic potato (272), tomato (273, 274), and rice plants (275, 276). Additionally, the TcpA antigen has also been expressed in transgenic tomato plants (277). However, transgenic rice expressing the CTX-B subunit has been by far the most studied.

In 2007, MucoRice-CTB, a transgenic rice-based vaccine expressing the CTX-B subunit, was developed. This platform produced an average of 30 μg of recombinant CTX-B per transgenic rice seed, which was stored in protein bodies (PBs), a type of storage organelle in rice. The *in vitro* assays with pepsin showed that the CTX-B was not degraded, suggesting that the PBs may act as a natural capsule for oral administration of the vaccine. Preclinical studies in mice and pigs orally immunized with the seed powder showed that MucoRice-CTB induced CTX-B-specific serum IgG and intestinal sIgA antibodies (275, 278–281). In the intestinal loop assay, the sIgA antibodies that were generated were found to confer protection against *V. cholerae* and LT-ETEC challenges (278). However, this formulation was not evaluated in an animal challenge assay to test whether it conferred protection against colonization by *V. cholerae*. This is probably because CTX-B-specific antibodies do not have vibriocidal activity. As a step toward the use of MucoRice-CTB in humans, this vaccine candidate was evaluated in non-human primates (*Macaca fascicularis*), inducing CTX-B-specific antibodies without adverse effects (279). Recently, a phase I study conducted in Japan showed that MucoRice-CTB increased CTB-specific serum IgG and IgA antibody levels without inducing serious adverse events. A similar phase 1 study is planned with individuals of other ethnicities (282).

## 9. Vaccine candidates in preclinical development

Some live OCVs were developed and evaluated in animal models several years ago, including IEM108 (283, 284), TLP01 (285), and VCUSM2 (286). However, no further related studies have been published since then. Although mentioned for historical reasons, interested readers are recommended to refer to earlier reviews where these vaccine candidates have already been discussed (287, 288).

Recent technological advances in vaccine design and manufacture have led to promising cholerae vaccine candidates, such as DuoChol^™^. This killed OCV is a lyophilized mixture of formalin-killed isogenic El Tor Ogawa and Inaba strains and rCTB in an enterocoated capsule. This formulation improves thermostability and could facilitate its integration into standard immunization programs in cholera-endemic areas. DuoChol^™^ is currently in preclinical development at the University of Gothenburg, Sweden (215, 227).

OMVs have emerged as a promising strategy for developing vaccines against Gram-negative bacterial pathogens, including *V. cholerae. V. cholerae* OMVs contain important virulence factors such as CTX, TcpA, OmpU, NanH, LPS, and others (70–72, 289, 290). Several preparations of OMVs derived from WT or mutant *V. cholerae* strains have been administered to mice through different routes, resulting in strong humoral responses against a variety of OMV-associated antigens. Immunization with OMVs protects against *V. cholerae* colonization regardless of the route of administration (291). In particular, intranasal immunization with OMVs induces O-specific antibodies, particularly IgG, which inhibit *V. cholerae* motility (292, 293). In another study, Leitner et al. (290) developed a combined formulation of OMVs derived from *V. cholerae* and ETEC. Interestingly, this OMV mixture conferred protection in mice against both pathogens, suggesting the potential for developing a broadly protective OMV-based vaccine against several Gram-negative pathogens by combining OMVs.

Virus-like particles (VLPs) are multi-protein structures that mimic the organization and conformation of native viruses, but lack the viral genome, making them a safe template for vaccine development (294). Over the past three decades, VLPs have served as a successful platform for developing vaccines against various viral diseases (295). However, their potential use against non-viral pathogens has scarcely been explored. A recent study reported the coupling of VLPs from the bacteriophage Qβ to the *V. cholerae* OSP antigen, which was immunogenic in mice, eliciting IgG antibodies with vibriocidal activity (296).

The development of chimeric proteins is a growing trend in the design of next-generation vaccines. The biotechnological revolution, particularly the improvements in gene synthesis, has opened new doors for the rational design of protein-based vaccines (297). Chimeric proteins carrying selected epitopes from several strains or different pathogens can enhance the immunogenicity of the recombinant antigen, eliciting a broader immune response (298). Chimeric protein-based vaccines against cholera have focused on known antigenic proteins, including the CTX-A and CTX-B subunits, flagellins, OmpW, OmpU, TcpA, TcpF, and NanH. Two vaccine candidates, TcpF-CTA2-CT-B and TcpA-CTA2-CT-B, are chimeric proteins (299, 300). Both chimeras were immunogenic in mice and triggered specific antibodies that conferred protection in passively immunized infant mice. However, no further studies have been published regarding these vaccine candidates. Similar results were obtained by the OTC chimera (OmpW, TcpA, and CTX-B), which elicited specific IgG antibodies that were protective in the ileal loop assay and in passively immunized infant mice (301).

An interesting recent study describes the polyvalent cholera MEFA protein, which contains antigenic domains of TcpA, CTX, NanH, HlyA, flagellins, and peptides mimicking the OSP on a flagellin B backbone (302). Mice and rabbits immunized intramuscularly with the MEFA protein developed antibodies to all the virulence factors targeted by the immunogen, except LPS. The antibodies generated neutralized CTX, bacterial motility, and *in vitro* adherence of *V. cholerae* O1, O139, and non-O1/non-O139 strains. Moreover, this vaccine provided cross-protective against *V. cholerae* O1, O139, and non-O1/non-O139 strains in adult and infant rabbit colonization models.

Despite promising results, protein-based vaccines have several limitations. For instance, they are often poorly immunogenic and require multiple doses and adjuvants to achieve protective immunity. In addition, they are generally administered parenterally to avoid enzymatic degradation in the stomach, inducing strong humoral responses at the systemic level but not at the intestinal mucosa level. Although this type of vaccine represents a potential alternative to OCVs, none of them have been tested in human trials. More importantly, they must compete in a market that demands cholera vaccines that are cost-effective and administered in a single-dose regimen.

## 10. Concluding remarks and prospects

Over the last few decades, much knowledge has been gained about the pathogenesis and immune response of *V. cholerae* infection, which has resulted in the development of treatments and vaccines. However, progress toward a highly effective cholera vaccine has been hindered by several limitations. These include the lack of a well-defined correlate of long-term protective immunity as well as an animal model that fully recapitulates the disease (303). In addition, it is largely unknown how the microbiota confers resistance or susceptibility to cholera and how it affects the immune response generated by vaccines against this disease (125). In this respect, human microbiota-associated mice could be a valuable animal model to consider (30).

Further studies are needed to investigate how immune responses are produced during *V. cholerae* infection. In particular, it is important to understand the innate immune pathways that are modulated during the natural course of infection, and whether these responses are beneficial or detrimental to the host. Additionally, it is crucial to clarify how long-term immune memory is generated in patients recovering from cholera. This information is essential because a highly effective cholera vaccine must recapitulate or mimic these immune responses.

OCVs have been shown to be safe, and although they confer short-term protection, their usefulness in cholera control has been reliably demonstrated. It is likely that new oral adjuvants, such as nanocarriers (304), lipid-based adjuvants (305), among other (306–308), could increase the efficacy of these vaccines.

The OMV-based vaccines, plant-based vaccines, and chimeric antigens are emerging and promising approaches in vaccine development. Moreover, mRNA vaccines against SARS-CoV-2 have been rapidly developed and have proven to be highly efficacious and adaptable as required. Recent studies have demonstrated the potential of mRNA vaccines against bacterial pathogens (309–311). Therefore, new cholera vaccine candidates based on these platforms are expected to appear in the coming years.

Another strategy to improve cholera vaccines could be the development of multivalent vaccines that protect against various enteric pathogens. Finally, in the human-pathogen arms race, the development of new vaccine technologies is likely the key factor in winning the battle and, ideally, in finding a highly long-lasting protective cholera vaccine.

## Author contributions

DM and RV wrote the draft of the manuscript. MO’R edited the manuscript. All authors contributed to data acquisition and analysis and approved the publication of the content.

## Funding

This work was supported by Postdoctoral FONDECYT grant 3190524, awarded to DM. Regular FONDECYT grant 1211647, awarded to RV.

## Conflict of interest

The authors declare that the research was conducted in the absence of any commercial or financial relationships that could be construed as a potential conflict of interest.

## Publisher’s note

All claims expressed in this article are solely those of the authors and do not necessarily represent those of their affiliated organizations, or those of the publisher, the editors and the reviewers. Any product that may be evaluated in this article, or claim that may be made by its manufacturer, is not guaranteed or endorsed by the publisher.
